# (Auto‑)Immunität bei fokaler Epilepsie: Mechanismen (auto‑)immun-inflammatorischer epileptogener Neurodegeneration

**DOI:** 10.1007/s00115-024-01695-5

**Published:** 2024-07-02

**Authors:** Nico Melzer, Katharina Weber, Saskia Räuber, Felix Rosenow

**Affiliations:** 1 Klinik für Neurologie, Medizinische Fakultät und Universitätsklinikum, Heinrich-Heine-Universität Düsseldorf, Moorenstraße 5, 40225 Düsseldorf, Deutschland; 2 Neurologisches Institut (Edinger Institut), Universitätsklinikum Frankfurt, Goethe-Universität Frankfurt, Frankfurt am Main, Deutschland; 3 Frankfurt Cancer Institute (FCI), Goethe-Universität Frankfurt, Frankfurt am Main, Deutschland; 4 Partnerstätte Frankfurt, Frankfurt am Main und Deutsches Krebsforschungszentrum (DKFZ), Heidelberg, Deutsches Konsortium für Translationale Krebsforschung (DKTK), Heidelberg, Deutschland; 5 Universitäres Centrum für Tumorerkrankungen Frankfurt (UCT), Universitätsklinikum Frankfurt, Goethe-Universität Frankfurt, Frankfurt am Main, Deutschland; 6 Epilepsiezentrum Frankfurt Rhein-Main, Klinik für Neurologie, Zentrum für Neurologie und Neurochirurgie, Universitätsklinikum Frankfurt, Goethe-Universität Frankfurt, Frankfurt am Main, Deutschland; 7 LOEWE Center for Personalized Translational Epilepsy Research (CePTER), Goethe-Universität Frankfurt, Frankfurt am Main, Deutschland

**Keywords:** Epilepsieentstehung, Immun-inflammation, Übererregbarkeit, Epileptoimmunologie, Immun-vaskulo-neurale Interaktion, Epilepsy development, Immune-inflammation, Hyperexcitability, Epileptioimmunology, Neuro-immune-vascular interaction

## Abstract

**Ziel der Arbeit:**

Während die neuronalen Mechanismen der epileptischen Übererregbarkeit („hyperexcitability“, HE) eingehend untersucht wurden, deuten neuere Erkenntnisse darauf hin, dass extraneuronale, hauptsächlich immun-inflammatorische und vaskuläre Mechanismen eine wichtige Rolle bei der Entwicklung und dem Fortschreiten der HE bei Epilepsie und ihren kognitiven und verhaltensbezogenen Begleiterkrankungen spielen.

**Material und Methoden:**

Narrativer Review.

**Ergebnisse:**

Auf der einen Seite können wie bei der autoimmunen (limbischen) Enzephalitis (ALE/AIE) oder der Rasmussen-Enzephalitis (RE) primäre adaptive und angeborene Immunantworten und damit verbundene Veränderungen der Blut-Hirn-Schranke (BHS) und neurovaskulären Einheit (NVU) selbst eine akute kortikale Übererregbarkeit (HE) verursachen und die Entwicklung einer Hippokampussklerose (HS) und andere strukturelle kortikale Läsionen mit chronischer HE hervorrufen. Auf der anderen Seite kann eine kortikale Übererregbarkeit, die bspw. mit Fehlbildungen der kortikalen Entwicklung (MCD) und niedriggradigen epilepsieassoziierten Tumoren (LEAT) assoziiert ist, begleitet sein von sekundären adaptiven und angeborenen Immunantworten und Veränderungen der BHS und NVU, wodurch möglicherweise deren Ikto- und Epileptogenität moduliert wird. Diese Zusammenhänge verdeutlichen den Einfluss adaptiver und angeborener Immunmechanismen und damit verbundener Veränderungen der BHS und der neurovaskulären Einheit auf die kortikale Erregbarkeit und umgekehrt, was für ein dynamisches komplexes Zusammenspiel dieser Faktoren bei der Entwicklung und dem Fortschreiten der Epilepsie im Allgemeinen spricht.

**Diskussion:**

Das geschilderte Konzept einer immun-vaskulo-neuralen Interaktion in der fokalen Epilepsie eröffnet neue Möglichkeiten des pathogenetischen Verständnisses und damit auch der selektiven therapeutischen Intervention.

## Abstract (en)

**Objective:**

While the neuronal mechanisms of epileptic hyperexcitability (HE) have been studied in detail, recent findings suggest that extraneuronal, mainly immune-mediated inflammatory and vascular mechanisms play an important role in the development and progression of HE in epilepsy and the cognitive and behavioral comorbidities.

**Material and methods:**

Narrative review.

**Results:**

As in autoimmune (limbic) encephalitis (ALE/AIE) or Rasmussen’s encephalitis (RE), the primary adaptive and innate immune responses and associated changes in the blood-brain barrier (BBB) and neurovascular unit (NVU) can cause acute cortical hyperexcitability (HE) and the development of hippocampal sclerosis (HS) and other structural cortical lesions with chronic HE. Cortical HE, which is associated with malformation of cortical development (MCD) and low-grade epilepsy-associated tumors (LEAT), for example, can be accompanied by secondary adaptive and innate immune responses and alterations in the BBB and NVU, potentially modulating the ictogenicity and epileptogenicity. These associations illustrate the influence of adaptive and innate immune mechanisms and associated changes in the BBB and NVU on cortical excitability and vice versa, suggesting a dynamic and complex interplay of these factors in the development and progression of epilepsy in general.

**Discussion:**

The described concept of a neuro-immune-vascular interaction in focal epilepsy opens up new possibilities for the pathogenetic understanding and thus also for the selective therapeutic intervention.

## Hintergrund

Nach Angaben der Weltgesundheitsorganisation (WHO) ist die Epilepsie eine der häufigsten schweren neurologischen Erkrankungen in Bezug auf Behinderung, Mortalität und verlorene Lebensjahre [61]. Epilepsie ist häufig mit kognitiven und psychobehavioralen Komorbiditäten verbunden, die stark zur Abnahme der Lebensqualität sowie der sozialen und wirtschaftlichen Teilhabe beitragen [61]. Derzeit leiden weltweit mehr als 50 Mio. Menschen an Epilepsie, und etwa ein Drittel der Patienten – meist mit fokaler Epilepsie – hat eine unzureichende Anfallskontrolle mit anfallssupprimierenden Medikamenten (ASM, pharmakoresistente Epilepsie; [37]). Zurzeit ist der einzige kurative Behandlungsansatz für diese Patienten die Epilepsiechirurgie [9, 69].

Zu den häufigsten Ursachen pharmakoresistenter fokaler Epilepsie, die durch Epilepsiechirurgie behandelt werden, gehören angeborene, statische Malformationen der kortikalen Entwicklung („malformations of cortical development“, MCD) einschließlich fokaler kortikaler Dysplasie („focal cortical dysplasia“, FCD) vom Typ I–III sowie erworbene niedriggradige epilepsieassoziierte Tumoren („low-grade epilepsy associated tumors“, LEAT) wie das Gangliogliom und die Hippokampussklerose (HS; [9]).

## Neuronale vs. extraneuronale Mechanismen der Anfalls- und Epilepsieentstehung

Eine Verschiebung der exzitatorisch-inhibitorischen Balance hin zu einer kortikalen Übererregbarkeit ist das Kernmerkmal der Epilepsie. Nicht nur das Kardinalsymptom – der epileptische Anfall (Iktus) –, sondern auch interiktuale epilepsietypische Entladungen (IED) in der Elektroenzephalographie (EEG) beruhen auf massiven, synchronisierten Entladungen von Neuronen, die in zerebralen Netzwerken organisiert sind, und sind ein wichtiger diagnostischer Biomarker für das Vorliegen einer Epilepsie. Die zugrunde liegenden Mechanismen und Konsequenzen kortikaler Übererregbarkeit bei Epilepsie sind weiterhin unvollständig verstanden. Während die neuronalen Mechanismen epileptischer Übererregbarkeit in den letzten Jahrzehnten ausgiebig untersucht wurden, deuten neuere Erkenntnisse darauf hin, dass extraneuronale, vor allem immuninflammatorische und vaskuläre Mechanismen eine wichtige Rolle bei der Entwicklung und dem Fortschreiten der kortikalen Übererregbarkeit bei Epilepsie und ihren kognitiven und psychobehavioralen Komorbiditäten spielen [6, 7, 36, 43, 55, 56, 68, 71].

Die Interaktion physiologischer mit epileptischen Netzwerken kann kognitive Leistung beeinträchtigen

Kognitive Komorbidität, die für die Lebensqualität der PatientInnen mit Epilepsie von hoher Relevanz ist, kann aus der Entstehung einer HS (Gedächtnisstörung) und anderen strukturellen kortikalen Läsionen resultieren, aber auch aus der Störung der physiologischen neuronalen Netzwerkaktivität durch Ausbreitung interiktualer und iktualer epileptischer Aktivität. D. h. die Interaktion physiologischer Netzwerke, die kognitive Funktionen unterstützen, mit epileptischen Netzwerken kann die kognitive Leistung beeinträchtigen, die von diesen Netzwerken vermittelt wird [1, 25, 30, 34, 65].

Diese komplexe, zeitlich wie örtlich dynamische Interaktion zwischen Immuninflammation und Dysfunktion der Blut-Hirn-Schranke (BHS) bzw. der neurovaskulären Einheit (NVU) in Bezug auf die Initiation und Progression der fokalen Übererregbarkeit in der Epilepsie eröffnet damit neue Zielstrukturen und -mechanismen für selektive, maßgeschneiderte Therapieinterventionen.

## Immun-vaskulo-neurale Interaktion bei der fokalen Epilepsie

Auf der einen Seite können wie bei der autoimmunen (limbischen) Enzephalitis oder der Rasmussen-Enzephalitis [16, 17, 28] *primäre *adaptive und angeborene Immunantworten und damit verbundene Veränderungen der BHS und NVU selbst eine akute kortikale Übererregbarkeit verursachen und die Entwicklung einer HS und andere strukturelle kortikale Läsionen mit chronischer HE hervorrufen [5–7, 24]. Auf der anderen Seite kann eine kortikale Übererregbarkeit, die bspw. mit MCD und LEAT assoziiert ist, begleitet sein von *sekundären* adaptiven und angeborenen Immunantworten und Veränderungen der BHS und NVU, wodurch möglicherweise deren Ikto- und Epileptogenität moduliert wird [5, 32, 36, 54, 71, 72]. Diese Immunreaktionen und die damit verbundene Veränderung der elektrischen Aktivität dieser Läsionen können zudem auch zur HS und anderen strukturellen kortikalen Läsionen („duale Pathologie“) führen [5–7, 24, 43, 68]. Kürzlich wurde in reseziertem epileptischem Hirngewebe von Patienten mit pharmakoresistenter fokaler Epilepsie eine proinflammatorische Mikroumgebung nachgewiesen, einschließlich einer umfangreichen Aktivierung von Mikroglia und der Infiltration anderer Immunzellen [36], sodass die Rolle sekundärer adaptiver und angeborener Immunantworten in solchen Läsionen zunehmend deutlich wird.

**Abb. 1:**
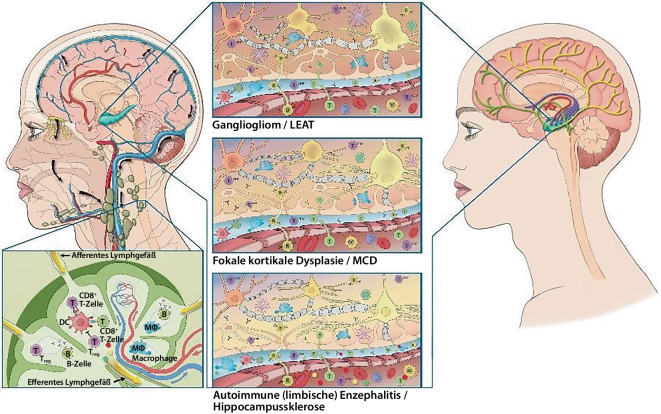
Interaktion von Übererregbarkeit, Immuninflammation und Blut-Hirn-Schranken-Störung bei fokalen Anfällen und Epilepsie am Beispiel der Temporallappenepilepsie. (Mit freundl. Genehmigung, © Heike Blum, alle Rechte vorbehalten.) *DC* dendritische Zellen, *LEAT* „low-grade epilepsy associated tumors“, *MCD* „malformations of cortical development“

Die möglichen Mechanismen, die der Initiierung und dem Zusammenspiel von Übererregbarkeit, Immuninflammation und Störung der BHS und NVU bei fokalen Anfällen und Epilepsie zugrunde liegen, sind in Abb. 1 dargestellt und werden im Folgenden diskutiert.

Ausgelöst durch periphere Tumoren [10, 48], Infektionen [12, 58] und/oder genetische Faktoren [50, 59] können bei der AIE/ALE oder der RE auf der einen Seite primäre adaptive und angeborene Immunantworten in den tiefen zervikalen Lymphknoten initiiert werden ([2, 14]; Abb. 1 unterer linker Bereich): Um eine adaptive humorale und zelluläre Immunantwort zu initiieren, die auf intrazelluläre oder auf der Zelloberfläche befindliche Antigene gerichtet ist, müssen sowohl professionelle antigenpräsentierende Zellen (APC) wie dendritische Zellen (DCs) als auch naive antigenspezifische B‑Zellen auf lösliche oder zellgebundene neurale bzw. neuralähnliche Antigene treffen und diese aufnehmen [48]. Beim Vorliegen defekter zentraler und/oder peripherer Immuntoleranzmechanismen [3, 19, 57, 67] können APCs diese Antigene dann im Zusammenhang mit Haupthistokompatibilitätskomplex(MHC)-Molekülen der Klasse II verarbeiten und naiven antigenspezifischen CD4^+^-T-Zellen präsentieren. Die MHC-Klasse-II-abhängige Erkennung durch diese CD4^+^-T-Zellen ist dann erforderlich, damit die B‑Zellen vollständig aktiviert werden können, um antikörpersezernierende Plasmazellen zu werden. Darüber hinaus können CD4^+^-T-Zellen APCs lizenzieren, um neurale Antigenpeptide im Kontext von MHC-Klasse-I-Molekülen zu präsentieren.

Naive antigenspezifische CD8^+^-T-Zellen erwerben zytotoxische Effektorfunktionen

Im Weiteren können dann naive antigenspezifische CD8^+^-T-Zellen aktiviert werden und zytotoxische Effektorfunktionen erwerben. Antikörpersezernierende Plasmazellen und zytotoxische CD8^+^-T-Zellen können zusammen mit Monozyten/Makrophagen aus den Lymphknoten austreten und über die BHS und NVU in das ZNS eindringen ([48]; Abb. 1 unteres mittleres Panel). In der Folge können diese Leukozyten eine humorale und zelluläre auf neurale Antigen gerichtete Autoimmunität im Hirnparenchym vermitteln ([48]; Abb. 1 unteres mittleres Panel): Glutamaterge Prinzipalneuronen und γ‑Aminobuttersäure (GABA)erge Interneurone können selektiv von antigenspezifischen CD8^+^-T-Zellen angegriffen werden aufgrund ihrer intrazellulären Antigenexpression und MHC-I-vermittelten Präsentation bspw. von Glutamat-Decarboxylase (GAD65) in Interneuronen [13, 22, 26, 44, 63] und Hu-Proteinen in Prinzipalneuronen [31] jeweils mit direkten Konsequenzen für die Netzwerkfunktion und Erregbarkeit.

In Bezug auf neurale Antigene an der Zelloberfläche kann die exzitatorische glutamaterge synaptische Übertragung und Plastizität durch Antikörper gegen N‑Methyl-D-Aspartat (NMDA) und α‑Amino-3-hydroxy-5-methyl-4-isoxazolepropionsäure(AMPA)-Rezeptoren gestört werden [15, 38, 45, 47]. Zudem kann die inhibitorische GABA(γ-Aminobuttersäure)erge synaptische Übertragung und Plastizität durch Antikörper gegen GABAA- und GABAB-Rezeptoren gestört werden [10, 27, 41, 53]. Antikörper gegen „leucin-rich glioma inactivated 1“ (LGI1) und „contactin-associated protein 2“ (CASPR2; [39, 40]) und andere neuronale und synaptische Adhäsionsmoleküle können sowohl die glutamaterge als auch die GABAerge synaptische Übertragung bzw. die intrinsische neuronale Erregbarkeit innerhalb des Netzwerks stören. Darüber hinaus kann die Fc-Rezeptor-Ligation durch Antikörper [52] und die Freisetzung proinflammatorischer Zytokine durch CD8^+^-T-Zellen [20, 35, 49] Mikroglia, Astrozyten und Makrophagen aktivieren, die wiederum zu synaptischer Dysfunktion und neuronalem Zellverlust beitragen. Darüber hinaus kann die antikörpervermittelte Aktivierung des Komplementsystems und bestimmter inflammatorischer Zytokine und Chemokine ebenfalls neuronale Degeneration verursachen und die neuronale Erregbarkeit verändern [8, 13, 21, 51, 62, 66, 70].

Diese Schritte der Transmigration von Immunzellen in das Hirnparenchym sind mit einer Beeinträchtigung der BHS und NVU verbunden, die bei entzündlichen Hirnerkrankungen wie Multipler Sklerose (MS; [18]) bereits gut untersucht sind. Die nachfolgende akute Übererregbarkeit kann zusammen mit zusätzlichen Veränderungen der BHS die Entwicklung von HS und anderen strukturellen kortikalen Läsionen mit chronischer Übererregbarkeit befördern [6, 7, 11, 23, 29, 43, 60, 64, 68].

MCD und LEAT können mit der Freisetzung (mutierter) neuraler Antigene einhergehen

Auf der anderen Seite können MCD und LEAT ([9, 36]; Abb. 1 obere Mittelteile) mit der Präsentation und Freisetzung (mutierter) neuraler Antigene einhergehen [33, 42]. Diese Antigene können über die gestörte BHS/NVU und das meningeale und glymphatische Abflusssystem [46, 56] in löslicher oder zellgebundener Form ([2, 4, 14]; Abb. 1 unterer linker Bereich) in die tiefen zervikalen Lymphknoten transportiert werden. Dort sind sie möglicherweise in der Lage aufgrund fehlender zentraler und peripherer Toleranzmechanismen gegenüber diesen Antigenen, eine sekundäre adaptive und angeborene Immunantwort auszulösen [4, 32, 36, 54, 72]. Antikörpersezernierende Plasmazellen und zytotoxische CD8^+^-T-Zellen können dann zusammen mit Monozyten/Makrophagen die aktivierte, gestörte BHS/NVU transmigrieren und in die epileptogene Läsion selbst gelangen (als Versuch, die darin enthaltenen aberranten Zellen zu eliminieren). Darüber hinaus beeinflussen diese Immunzellen und ihre Effektormoleküle möglicherweise auch intakte neuronale Strukturen, die an der weiter ausgebreiteten interiktualen und iktualen epileptischen Aktivität, d. h. der irritativen und epileptogenen Zone, beteiligt sind. Sie modulieren dadurch möglicherweise die räumliche Ausdehnung dieser Bereiche ([4, 32, 54, 72]; Abb. 1 obere Mittelteile). Da sich die meisten MCD und LEAT im Temporallappen befinden, kann die Ausbreitung der epileptischen Aktivität und möglicherweise auch der Immunantwort zur HS und anderen strukturellen kortikalen Läsionen führen („duale Pathologie“; [23, 43, 60, 64, 68]).

## Fazit für die Praxis

- Die dargelegten Überlegungen unterstreichen den Einfluss lokalisierter adaptiver und angeborener Immunmechanismen und damit verbundener Veränderungen der Blut-Hirn-Schranke (BHS) und der neurovaskulären Einheit (NVU) auf die fokale kortikale Erregbarkeit und umgekehrt, was auf ein dynamisches komplexes Zusammenspiel zwischen diesen Faktoren in der Ikto- und Epileptogenese hindeutet, die einen umschriebenen kortikalen Bereich sowohl auf zellulärer und molekularer als auch auf der Netzwerkebene beeinflusst. Die strukturellen und funktionellen Auswirkungen dieser lokalisierten Prozesse können sich über Verbindungen zu anderen Hirnarealen ausbreiten und so weit verbreitete neuronale Netzwerke stören, was zu kognitiven und psychobehavioralen Komorbiditäten auf der Systemebene führt.
- Das geschilderte Konzept einer immun-vaskulo-neuralen Interaktion in der fokalen Epilepsie eröffnet neue Möglichkeiten des pathogenetischen Verständnisses und damit auch der selektiven therapeutischen Intervention.
